# Physicians reading and writing practices: a cross-sectional study from Civil Hospital, Karachi, Pakistan

**DOI:** 10.1186/1472-6947-12-76

**Published:** 2012-07-27

**Authors:** Muhammad Farhan Khaliq, Muhammad Muslim Noorani, Uzair Ahmed Siddiqui, Maheen Anwar

**Affiliations:** 1 Dow Medical College, Dow University of Health Sciences, Civil Hospital, 203 Shan Plaza, Gawali Lane No. 3, Ratan Talao Near Urdu Bazar, Karachi, Pakistan

**Keywords:** Sources of information, Up to date, Research, Literature reading, Journal club, Developing country, Pakistan, Karachi, Civil hospital

## Abstract

**Background:**

To determine the behavior of physicians regarding medical literature reading and participation in research activities at one of the largest teaching hospitals in Pakistan.

**Method:**

This descriptive, cross-sectional study was conducted by interviewing the house officers, residents and fellows of six major specialties (Medicine, Surgery, Pediatrics, Psychology, Obstetrics & Gynecology and Anesthesia) in Civil Hospital, Karachi between August and December, 2011. The questionnaire elicited responses regarding the reading habits of physicians, preferred sources of information, their participation in research activities (publication & supervision) and views regarding journal club. SPSS 17.0 was used for data entry and analysis.

**Result:**

A total of 259 completely filled questionnaires were returned with a response rate of 85.19%. Mean age of the participants was 29.67 ± 7.65 years. Books were selected by 71.4% doctors as their preferred source of information, regardless of their clinical specialties. (p < 0.05). E-journals were preferred by 75.7% of the doctors over printed journals. This holds true for doctors from all specialties (p < 0.05). The ease of searching for relevant articles was the major contributor (50.5%) in preference of e-journals. 137 (52.9%) doctors read 5 or less articles per week. 30 (11.6%) doctors have subscription of journals (printed or electronic). At least one research paper has been published by 151 (58.3%) of the physicians interviewed. Most common reason for not participating in research activities was busy schedule (56.4%). Almost half (49.4%) doctors reported lack of journal club in their units. Of these, majority (88.35%) wanted a journal club in their respective units.

**Conclusion:**

Urgent intervention is required to promote healthcare literature reading and writing practice in our physicians. Easy access to workplace computers with internet and subscription of paid journals will facilitate physicians. Lack of supervisors and busy schedule were reported to be important contributors for not participating in research. Addressing these issues will encourage doctors to participate more in research activities.

## Background

The last few decades have witnessed a tremendous upsurge in biomedical research. It is estimated that 6-7% of new information is inculcated per annum, doubling the information in ten to fifteen years [1]. These facts necessitate thorough scrutiny of biomedical research before it can be used to alter the existing medical practice.

The literature reading habits of doctors are influenced by various factors such as the professional age, location and size of their primary hospital, the type of practice and the specialty to which they belong [2]. The time limitations imposed by a hectic schedule warrants clinicians to study medical literature based on practical relevance [3,4].

Reading and writing medical literature contributes to the development of critical thinking which is an important trait for the practicing clinicians [5]. Unfortunately, in Pakistan, doctors have attached more emphasis towards clinical practice with little input in the field of research [6]. The causes implicated for this trend are lack of time and infrastructure, financial constraints, lack of future benefits and paucity of mentors who are well-versed in research [7]. In the recent years, however, efforts have been made to inculcate research culture into clinical practice with the requirement of having two publications in journals indexed by Index Medicus, by College of Physicians and Surgeons, Pakistan (CPSP) [8]. Publishing articles has also been made mandatory by CPSP for doctors, for ascending to the next level in tenure system. The recent years have also witnessed increased involvement of medical students and fresh graduates towards medical research [9].

There is a very little literature which represents the reading habits of doctors of public sector hospitals in Pakistan. This study was carried out to observe what measures doctors take to keep themselves updated with new medical literature and their involvement in research activities.

## Methods

### Study setting

This descriptive study was conducted between August to December 2011 at Civil Hospital Karachi (CHK) which is a 1900 bed tertiary health care centre. It is one of the largest teaching hospitals of Pakistan where more than 1400 doctors are employed as house officers, residents and fellows in 21 specialties.

### Study population and data collection

The sample size was calculated with the assumption that 50% of doctors read medical literature and are involved in research activities. A sample size of 304 was obtained at 95% confidence interval and 5% margin of error. For the study sampling, the 21 specialties were sorted into 6 major groups. These groups were categorized as Medicine (which included Internal Medicine, Dermatology, Neurology and Cardiology) Surgery (which included General Surgery, Neurosurgery, Ophthalmology and Otolaryngology), Pediatrics, Anesthesiology, Gynecology & Obstetrics and Psychiatry. The study protocol was reviewed by the Institutional Review Board of Dow University of Health Sciences.

The questionnaire was constructed after a pilot study in which 30 random doctors were included. These doctors were provided with open ended questionnaires. The results of these questionnaires were analyzed and were used to design it. should be: It also included pertinent questions as indicated by other studies and suggestions from faculty members of community medicine department. Written consent was obtained from participating doctors and a self-administered questionnaire was provided to them. The study participants were assured of the confidentiality of their data. The participants were requested when they were given the questionnaires, to complete and return them at the same moment. When this was not possible, cell phone numbers of participants were noted and the forms were collected from them later. A database of the participants was constructed which included the name of participant, their respective department and serial number of the form of participant.

### Study instrument

The self-administered questionnaire consisted of five sections. Section one dealt with participant demographics. It had questions regarding age, education level (house officer, post-graduate trainee or training completed), teaching status and their unit. Section two consisted of questions regarding reading habits of participants. This sections had questions regarding sources of information used, literature reading frequency and language preference (English or native language). Preference for journal content was asked. The participants were also questioned about the location where they studied literature. Another question aimed to determine reasons of not reading literature from those who had indicated this in their responses.

Section three dealt with e-journals. The participants were asked whether they preferred e-journals over printed journals and reasons were elicited for the same. They were asked about the sources of literature they read online and the last time they searched for an article over the internet. Section four investigated the research related activities of the participants. It had questions about the number of articles each participant had published and supervised. Those participants who were not involved in any research related activity, were asked the reason for being so. Section five dealt with determining journal club activity in units of participants. In those units which had no journal club activity, participant opinion on starting one was asked.

### Statistical methods

Statistical Package for Social Sciences Windows version 17 was used for database assembly and analysis. Only those questionnaires were included which were completed. Descriptive analysis (means, standard deviations and percentages) was performed. To determine significant associations between variables, cross-tabbing of the variable was performed and Pearson Chi squared test was applied. Values were considered significant when they were below 0.05 (p < 0.05).

## Results

### Response rate

The authors collected 259 completed questionnaires from the sample size of 304 doctors. In addition to this, six incomplete questionnaires were also received which were excluded from the study. This yields a response rate of 85.19%.

### Respondent characteristics

Of the returned questionnaires, 122 (47.1%) were filled by males whereas 137 (52.9%) by females. The mean age of the participants was 29.67 ± 7.65 years. The study sample population comprised of 138 house officers (53.3%), 78 residents (30.1%) and 43 fellows (16.6%). Most respondents belonged to Medicine (35.1%), and Surgery (21.6%). Other doctors were from Pediatrics (12.7%), Anesthesia (12%), Psychiatry (10%) and Obstetrics and Gynecology (8.5%).

### Sources of information

**Figure 1:**
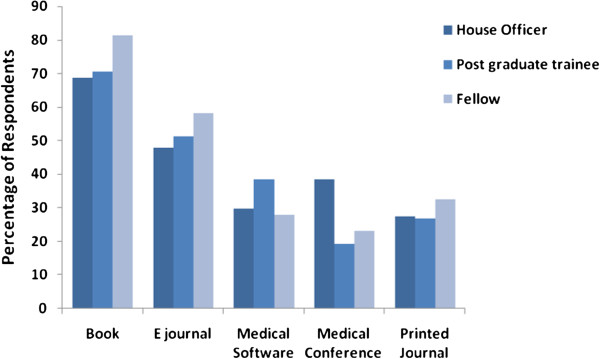
Sources of Information used by Pakistani Physicians.

The source of medical information, which is used most commonly, is medical books. This was affirmed by 185 (71.4%) doctors. 131 (50.6%) doctors answered in positive about reading e-journal to keep themselves updated. (Please see Figure 1). Less important sources were medical software, medical conferences and printed journals which were reported by 83 (32%), 78 (30.1%) and 73 (28.2%) doctors, respectively. (Please see Table 1 for specialty wise breakdown of information sources).

**Table 1:** Relationship between physician specialties and reading preferences

		**MEDICAL SPECIALTIES**						
		**Medicine**	**Surgery**	**Gynecology**	**Pediatrics**	**Anesthesia**	**Psychiatry**	
		** n (%)**	** n (%)**	** n (%)**	** n (%)**	** n (%)**	** n (%)**	**P values**
**Preference of Electronic journals over Printed journals**		77 (84.6)	51 (91.07)	21 (95.45)	18 (54.54)	16 (51.6)	13 (50)	**<0.001**
**Paid Subscription of journals**		12 (13.18)	11 (19.64)	-	2 (6)	5 (16.1)	-	**0.03**
**Books**		72 (79.12)	34 (60.71)	11 (22)	17 (51.5)	28 (90.3)	23 (88.46)	**<0.001**
**E journals**		35 (38.46)	40 (71.42)	7 (31.8)	20 (60.6)	17 (54.8)	12 (46.15)	**0.001**
**Medical Software**		25 (37.87)	20 (35.71)	9 (40.9)	11 (33.3)	10 (32.2)	8 (30.76)	**NS**
**Printed Journals**		30 (32.9)	15(26.78)	7 (31.8)	6 (18.18)	8 (25.8)	7 (26.9)	**NS**
**Medical Conferences**		32 (35.16)	18 (32.14)	8 (57.14)	8 (24.24)	5 (16.1)	7 (26.9)	**NS**
**Articles ** **read per week**	**None**	44 (48.3)	3 (5.3)	7 (31.8)	11 (33.3)	14 (45.1)	7 (26.9)	**<0.001**
	**<5**	38 (41.7)	38 (67.8)	10 (45.4)	22 (66.6)	12 (38.7)	17 (65.3)	
	**<10**	7 (7.6)	11 (90.6)	5 (22.7)	-	2 (6.4)	1 (3.8)	
	**<15**	2 (2)	4 (7.1)	-	-	2 (6.4)	1 (3.8)	
	**More than 15**	-	-	-	-	1 (3.2)	-	

*p < 0.05 considered significant.

*n (%) are from those physicians who gave a positive response to that particular question.

In terms of literature reading frequency, 137 (52.9%) doctors read less than 5 articles per week. A reading frequency of 6–10 articles per week was reported by 26 (10%) doctors. Whereas 9 (3.5%) doctors confirmed reading 11–15 articles per week. Only one (0.4%) doctor reported reading more than 15 articles in a week. 86 (33.2%) reported reading zero articles per week. (Please see Table 1).

**Figure 2:**
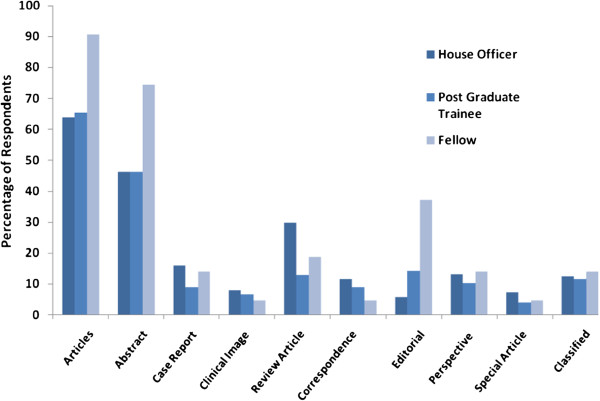
Journal Sections read by Pakistan Physicians.

Regarding components of journals, 178 (68.7%) read articles in the journals. Abstracts of articles are read by 132 (51%) of the doctors. 59 (22.8%) reported reading review articles in the journals. Other sections of the journals including case reports, perspective articles, editorial, correspondence, clinical image, special features and classified were read by less than 20% of the respondents. (Please see Figure 2). Subscription of medical journals, either online or in print, was confirmed by only 30 (11.6%) doctors. Most popular journals amongst our study population were Journal of Pakistan Medical Association (63.2%) and Journal of College of Physicians and Surgeons Pakistan (60.1%).

The age of the participants was found to be significantly related with articles read per week, paid subscription to journals and e-journals, medical software and medical conferences as modes of information (p < 0.05). Gender of participating doctors was found to be significantly related with using books as source of information, articles read per week (p < 0.05). The teaching faculty status of the participating physicians was found to be significantly related to articles read per week and paid subscription to journals (p < 0.05).

#### Electronic journals

E-journals were preferred over printed journals by 196 (75.7%) doctors. This question was a multiple response item where subjects were asked to check all items which they found to be relevant with their choice of e-journals over printed journals. The primary reason for such preference was that articles of relevance can be easily searched as reported by 50.5% doctors. Ease of reading articles was cited to be the reason for the choice of e-journals by 29% participants. Economical use of time (29%), remote access to articles away from the library (28.5%) and keeping up with day to day changes in trends in medicine (22.4%) were other reasons for doctor’s preference of e-journals.

In our study population, 110 (47.6%) doctors did not prefer e-journal over their printed counterparts. The questionnaire aimed to elicit reasons for this attitude of our study participants. The most common reason mentioned was subscription charges (77.7%). Busy schedule was indicated as a participating cause in this attitude by 58.7% clinicians. Lack of interest was quoted as a cause by 31.7% study participants. Connectivity issues to the internet trouble 20.6% of clinicians. (Please see Table 2).

**Table 2:** Hierarchical dissection of physician preferences and activities

	**House Officers n (%)**	**Residents n (%)**	**Fellows n (%)**
**Preferred E journals over Printed Journals**	116 (84)	57 (73)	23 (53.4)
Reasons*			
Easier to read	30 (35.8)	21 (36.8)	6 (26)
Articles of choice	63 (54.3)	29 (50.8)	7 (30.4)
Latest trends	24 (20.6)	18 (31.5)	2 (8.6)
Less time consuming	32 (27.5)	19 (33.3)	6 (26)
Can Access from any where	34 (29.3)	21 (36.8)	1 (0.3)
**Didn’t prefer e-journals over printed**	22 (15.9)	21 (26.9)	20 (46.5)
Reasons**			
Subscription charges	12 (54.5)	18 (85.7)	19 (95)
No connectivity	3 ( 13.6)	7 (33.3)	3 (15)
Busy schedule	10 (45.4)	14 ( 66.6)	13 (65)
Not interested	6 (27.2)	6 (28.5)	8 (40)
**Articles Published**			
None	43 (31.1)	35 (44.8)	16( 37.2)
Less than 5	89 ( 64.4)	43 (55.1)	19 (44.1)
5 - 10	6 (4.3)	-	4 (9.3)
More than 10	-	-	4 (9.3)
**Articles Supervised**			
None	79 (57.2)	43 (55.1)	24 (43)
Less than 5	59 (42.7)	35 (44.8)	15 (34.8)
5 or greater	-	-	4 (9.3)
No articles Published***			
**Reasons**			
Busy Schedule	18 (41.8)	24 (68.5)	11(68.7)
Not acquainted with research methodology	26 (60.4)	15 (42.8)	6 (37.5)
Lack of Interest	12 (27.9)	16 (45.7)	7 (43.7)
Lack of incentive	12( 27.9)	17 (48.5)	10 (62.5)

*Percentage calculated from those who preferred e-journals over printed.

**Percentage calculated from those who did not prefer e journals over printed.

***Percentage calculated from those who haven't published any research article.

Regarding place where doctors study literature over the internet, 202 (78%) respondents chose home as the primary place of study. In tertiary care hospital, 54 (20.8%) of doctors browse literature online. Cell phone/smart phone/PDA usage for this purpose was reported by 32 (12.4%) doctors. 9 (3.5%) doctors reported reading literature online at some other place of work. Search engines of Google Scholar (87.6%), US National Library of Medicine (Pubmed) (47.9%) and Pakmedinet (23.6%) were the sources most used by doctors for searching articles on the internet. For searching medical related topics, 157 (60.6%) doctors reported using the internet in the past week.

We found significant association between age, gender and teaching status of the participating physicians with preference for e-journals (p < 0.05).

### Participation in research

In our study population, 94 (36.3%) doctors reported publishing no article in a medical journal, at the time of interview. 151 (58.3%) doctors reported publishing less than 5 articles whereas 14 (5.4%) doctors confirmed that they had published 5 or more articles.

Of the 94 doctors who had not published any article at the time the study was conducted, 43 (45.7%) were house officers, 35 (37.2%) were residents and 16 (17%) were fellows. When subgroup analysis was done in relation with education of the participants, it yielded 31.1%, 44.8% and 37.2% respective percentages for house officers, residents and fellows. This relationship between education level and no published articles was found to be significant.

Busy schedule was indicated as the most pressing reason for not participating in research activities by 56.4% doctors who hadn’t published any research work. Lack of acquaintance with research methodology was reported as a hindrance by 50% doctors. Lack of interest and lack of incentive were the reasons cited by 37.2% and 41.4 clinicians respectively.

When the role of doctors as supervisors in articles was assessed, 146 (56.4%) doctors hadn’t supervised any article, at the time of interview. 109 (42%) clinicians had supervised less than 5 articles, whereas 4 (1.5%) had supervised 5 or more articles. We found significant relationship between the age of the participants and articles published by them (p < 0.05). Furthermore, a significant relationship was observed between teaching status of participants and publication and supervision of research articles (p < 0.05).

#### Journal club

The presence of journal club in their respective wards was affirmed by 131 (50.6%) doctors. Of the remaining 128 (49.4%) doctors, more than 85% doctors in each of the 6 categories (medicine, surgery, obstetrics & gynecology, pediatrics, anesthesia and psychiatry) would like to attend journal club should there unit decide to start one. When unit level analysis was performed, it was found that 92% participants in medicine, 87% in surgery, 86% in obstetrics & gynecology, 87% in pediatrics, 89% in anesthesia and 86% in psychiatry would like to attend journal club in their respective units.

## Discussion

Our study highlights that text books are the primary source of information of doctors. This preference is independent of the level of education and the hierarchical placement of doctor in the hospital system. Studies conducted in the USA show a similar trend of devotion towards books [10-13]. E-journals were rated as the second most preferred source for information by our clinicians. More than 50% of residents responded positively about reading e-journals. This indicates a positive attitude of our clinicians towards incorporating more recent advances in medicine in their training. Most of our study respondents (68.7%) reported reading full text articles from the journals. This particular choice of reading material is imperative for development of proper understanding regarding issues of clinical importance [14].

Comparison of printed versus electronic journal revealed a preference for the latter from more than 75% of the physicians interviewed. Similar findings have been reported in studies conducted elsewhere [15-17]. The ease of locating relevant articles from the electronic databases was suggested as the most common reason for choosing the electronic form over paper. This indicates that despite busy schedules, doctors take measures to keep abreast with the on-going medical developments. Most of the doctors interviewed reported accessing articles from their homes. This reflects the lack of quick reference practice using smart phones and personal digital assistants (PDA)’s by our doctors. This is in contrast to the practices of medical students and residents in USA who use their PDA’s and software for practicing evidence based medicine [18,19]. The use of telecommunication facilities for acquiring knowledge for evidence based medicine does not appear to parallel the growth of this sector in Pakistan.

Nearly 25% of our study population refrained from using electronic journals. High subscription charges of journals was the reason held responsible by most (77.7%) of the doctors who were reluctant to browse electronic journals. Unavailability of time was regarded an impediment for usage of electronic literature by 58% of the respondents. 56.4% of our study participants suggested that hectic schedule as the hindrance for non-participation in research related activities. This was followed by lack of acquaintance with research methodology for no publications (50%).

In our setup, research training is still at grass roots level with very few mentors for a large student population. Aslam, F. reports that majority of articles (almost 95%) in the student corner of the Journal of Pakistan Medical Association are contributed by students of private medical colleges [20]. This is food for thought for the policy makers at public medical schools as inculcating research training at medical school level is essential for training more research oriented doctors for tomorrow [21]. One can see the benefits of the recent initiative of introducing research training in medical curriculum in our study where 68.8% of house officers have reported to publish some research article.

Journal critique club is a well established method of acquainting physicians with latest development [22-25]. This not only serves the purpose of enhancing their clinical knowledge, but also raises their interest about research activities. This critique also nurtures the ability of critical appraisal of different scenarios and helps to develop better insight in the clinicians. Almost half the doctors interviewed pointed out that there were no journal club meeting in their workplace. However, about 88.3% of these doctors are keen in participating in such an activity, if it were to be commenced in their ward.

When our results were compared with other studies with similar interest conducted in developing countries, several disparities were noted. Doctors from Kenya and Egypt reported high interest in reading articles from North American journals which impact their clinical practice [26]. In contrast to this finding, our participants were more inclined towards local journals. Due to high subscription charges of journals, most doctors cannot afford to read them if there is no institutional support, as in our case.

In a study from Nigeria, Ajuwon G states that Nigerian physicians are proficient in using Internet for patient healthcare (more than 90% replied in affirmative about different domains in their questionnaire), with rates being even higher than those in Switzerland and USA in some cases [1]. When our results were compared with these, they were found to be a lot less than of doctors in Nigeria. The lack of internet connection at primary place of work is the problem mentioned by both Pakistani and Nigerian doctors. However, the Nigerian doctors access the internet through cyber cafés whereas our doctors used residential internet connections. The difference that exists between the two populations needs to be bridged. A proposed solution is journal club initiative in all units and mandatory attendance in such sessions. This would serve to increase the exposure of doctors to internet for searching healthcare related literature.

We see a tepid response our physicians towards research activities. There are several reasons for such lukewarm response. Foremost amongst them is the total absence of a formal training in their medical school only until recently. Previous studies conducted in Pakistan and India have already highlighted the importance of absence of formal training in research methodology and biostatistics [27,28]. This no doubts impacts the future response of doctors towards research activities. Other noteworthy factors for this behavior are too few experienced supervisors, no or ineffective journal club activities and lack of institutional support for subscription to paid journals which promote interest in research. Where these factors are mentioned, it is essential to mention, that most studies conducted recently in our setup have been cross sectional studies which contribute less towards solving the problem at hand, as compared to clinical trials [2,29]. Lack of funding plays a key role in preference for cross-sectional studies.

### Limitation

The convenience sampling employed in this study and the limitation of this study within a single centre; limit its generalization to the entire doctor population of Pakistan.

The study sample was limited to practicing clinicians from the hospital. It excluded private practitioners as well as doctors related to basic sciences without clinical practice. It is likely that trends differ in these groups. Self reporting and a low response rate (as compared to similar studies) are also the limitations of this study.

## Conclusion

The medical literature reading and writing practices of our physicians plead for an urgent intervention from concerned authorities. This can range from support provided through institutional subscription of various journals to establishment of digital libraries with high speed internet connection and online subscription to journals. There is also a need for provision of medical software and gadgets which enable our doctors to remain in touch with updated healthcare literature while on the move.

To promote research culture in future doctors, we propose that research training at the medical school level be reinforced with mentor system. This should take into account individual student interest to make it more effective. Changes in syllabus are necessary to help students develop a definitive and concrete understanding of biomedical research terminology and how to interpret researches. This understanding of biomedical research should also be furthered at the resident level with journal club meetings and small focus groups held within wards. Attendance in such sessions should be made compulsory to ensure that proper understanding is acquired by all participants. Evidence based medicine keeps in consideration the regional difference in etiologies of diseases. Hence the need for local data from our population to guide therapy effectively cannot be emphasized enough. This requires periodic reminders to doctors and medical students to take part in research activities. Appreciation of research work should be made a part of institutional mindset. This will go a long way in ensuring that doctors are motivated to produce more original research work with special focus towards regional problems.

## Competing interest

The authors declare that they have no competing interests.

## Authors’ contribution

MFK and MMN designed the study. The work of Data collection and database construction was distributed equally amongst authors. Data analysis and interpretation was done by MFK, MMN and UAS. Provisional drafts of manuscript were written by MFK, MMN, UAS and MA. Final revisions of draft were done by MFK and MMN. All authors read and approved the final manuscript.

## Pre-publication history

The pre-publication history for this paper can be accessed here:

http://www.biomedcentral.com/1472-6947/12/76/prepub
